# First study on *in vitro* antiviral and virucidal effects of flavonoids against feline infectious peritonitis virus at the early stage of infection

**DOI:** 10.14202/vetworld.2023.618-630

**Published:** 2023-03-26

**Authors:** Chanittha Triratapiban, Varanya Lueangaramkul, Nantawan Phecharat, Achiraya Pantanam, Porntippa Lekcharoensuk, Sirin Theerawatanasirikul

**Affiliations:** 1 Department of Microbiology and Immunology, Faculty of Veterinary Medicine, Kasetsart University, Bangkok 10900, Thailand; 2 Center for Advanced Studies in Agriculture and Food, Kasetsart University Institute for Advanced Studies, Kasetsart University, Bangkok 10900, Thailand; 3 Department of Anatomy, Faculty of Veterinary Medicine, Kasetsart University, Bangkok 10900, Thailand

**Keywords:** antiviral, feline coronavirus, feline infectious peritonitis virus, flavonoids, infectious disease

## Abstract

**Background and Aim:**

Feline infectious peritonitis (FIP), one of the most important infectious diseases in cats is caused by FIP virus (FIPV), a mutated variant of feline coronavirus. Feline infectious peritonitis has a negative impact on feline health, with extremely high mortality in clinical FIP-infected cats, particularly young cats. There are no approved drugs for FIP treatment, and therapeutic possibilities for FIP treatment are limited. This study aimed to utilize nature-derived bioactive flavonoids with antiviral properties to inhibit FIPV infection in Crandell–Rees feline kidney (CRFK) cells.

**Materials and Methods:**

The cytotoxicity of 16 flavonoids was evaluated on CRFK cells using a colorimetric method (MTS) assay. Viral kinetics of FIPV at 50 tissue culture infectious dose (TCID_50_)/well was determined during the first 24-h post-infection (HPI). Antiviral activity was evaluated based on the replication steps of the virus life cycle, including pre-compound, attachment, penetration, post-viral entry, and virucidal assays. The antiviral efficacy of flavonoids against FIPV was determined based on positive FIPV-infected cells with the immunoperoxidase monolayer assay and viral load quantification using reverse transcription-quantitative polymerase chain reaction.

**Results:**

Two flavonoids, namely, isoginkgetin and luteolin, inhibited FIPV replication during post-viral entry in a dose-dependent manner, with 50% maximal effective concentrations = 4.77 ± 0.09 and 36.28 ± 0.03 μM, respectively. Based on viral kinetics, both flavonoids could inhibit FIPV replication at the early stage of infection at 0–6-HPI for isoginkgetin and 2–6-HPI for luteolin using a time-of-addition assay. Isoginkgetin exerted a direct virucidal effect that reduced the viral titers by 2 and 1.89 log_10_ TCID_50_/mL at 60 and 120 min, respectively.

**Conclusion:**

Isoginkgetin interfered with FIPV replication during both post-viral infection and virucidal experiments on CRFK cells, whereas luteolin inhibited the virus after infection. These results demonstrate the potential of herbal medicine for treating FIP.

## Introduction

Feline infectious peritonitis (FIP) is a fatal infectious disease in young cats caused by FIP virus (FIPV), a mutated virulent feline coronavirus (FCoV) biotype. Feline infectious peritonitis virus belongs to the genus *Alphacoronavirus*, family *Coronaviridae*, which is grouped within the same family as severe acute respiratory syndrome CoV (SARS-CoV) and SARS-CoV-2 [1, 2]. Feline CoV is grouped into two biotypes based on pathobiology. Feline enteric CoV (FECV), an avirulent strain, is ubiquitous in cat populations. Feline enteric CoV mainly replicates in the enteric epithelium and causes mild enteritis and subclinical symptoms. Feline infectious peritonitis virus is a virulent strain, which changes tropism from intestinal cells to macrophages or monocytes due to spontaneous mutations within the viral genome [2–4]; therefore, FECV-infected cats develop FIP. Feline infectious peritonitis virus causes a severe systemic and fatal disease in young cats, which is characterized by fibrinous and granulomatous lesions with or without protein-rich serous effusions in body cavities [1–3]. Pregnant cats may have fetal resorption and abortion [4]. Although FIP vaccines have been developed, the efficacy of available FIPV vaccines is limited [1, 2]. Unfortunately, specific therapeutic and prevention strategies are not available, but the demands for FIP therapy from veterinary practitioners and cat owners are increasing. Therefore, the need for antiviral therapy in FIPV-infected cats must be met.

Studies have investigated the effectiveness of antiviral agents that inhibit viral replication *in vitro* as potential drugs. However, effective drug doses are often cytotoxic; a bottleneck in translating *in vitro* findings to clinical cases. High-potential anti-FIPV agents, such as GC376 (call as GC) [5, 6] and GS-441524 (call as GS) [7–9], have been used for treating FIP. However, the administration of both agents through S/C injection every 12–24 h for up to 120 d caused severe tissue irritation and pain at the injection site. Cats cannot tolerate the injectable form of GS. Moreover, high dosages are required for drug distribution throughout the body [5–10]. Both GC and GS are unlicensed drugs; hence, they are sold in the black market on the internet at a great cost [9, 10]. Many concerns remain about the widespread use of unapproved FIP treatments that may cause minor mutations in the viral genome, thereby leading to drug resistance. The use of a pro-drug of GS-441524 (GS-5734; Remdesivir) led to two mutations of murine hepatitis virus nsp12 at the conserved residues across CoVs [11]. In addition to unapproved drugs, small-molecule inhibitors have been investigated to treat FIPV infection by focusing on each step of the viral cycle. ERDRP significantly inhibited the replication of FIPV in a dose-dependent manner [12]. Some FDA-approved drugs have been investigated *in vitro* for repurposing application to combat FIPV infection. For instance, chloroquine, mefloquine, and hexamethylene amiloride [13] and itraconazole could reduce FCoV-I replication [14]. Nature-derived phytochemicals from plants, animals, fungi, and marine products possess antioxidant, anti-inflammatory, anti-cancer, and antiviral activities [15, 16]. Recently, the development of drugs using plant-based bioactives or crude extracts for combination therapy has increased. Flavonoids are a group of secondary metabolites produced by many plants [16]. These phytochemicals have many beneficial effects, including antiviral activities [15, 16]. Traditional herbal medicines and purified natural products may guide the development of novel antiviral drugs. Several antiviral mechanisms have been reported, and nature-derived products have been identified as potent FIPV inhibitors [13, 17]. Flavonoids can interact with various steps of viral infection, including viral attachment, penetration, and post-viral infection. Reports on the antiviral activity of flavonoids in veterinary medicine are limited. Feline infectious peritonitis virus infection in cats is considered a model for studying antiviral agents against CoVs in humans and animals [18].

This is the first study on the inhibitory effects of bioactive flavonoids on FIPV in Crandell–Rees feline kidney (CRFK) cells. Antiviral activity was determined using a cell-based assay, and the effectiveness was evaluated using immunostaining and reverse transcription-quantitative polymerase chain reaction (RT-qPCR). The findings of this study give insights into the use of flavonoids as anti-FIPV agents *in vitro*.

## Materials and Methods

### Ethical approval

Ethical approval is not necessary for this type of study.

### Study period and location

The study was conducted from March 4^th^, 2021, to September 1^st^, 2022. All experiments in this research were carried out in the Virology Laboratory at Department of Microbiology and Immunology, Faculty of Veterinary Medicine, Kasetsart University, Thailand. The procedures involved FIPV were performed under a biosafety cabinet in the BSL-2 laboratory to comply with the biosafety and biosecurity regulations.

### Cell lines and virus

Crandell–Rees feline kidney cells (CRFK; ATCC^®^, Manassas, VA, USA) were cultured in modified Eagle’s medium (MEM; Gibco™ Thermo Fisher Scientific Inc., Waltham, MA, USA) with 10% (v/v) heat-inactivated fetal bovine serum (FBS; Gibco™ Thermo Fisher Scientific Inc.). Cells were maintained at 37°C in a humidified incubator with 5% CO_2_. The FIPV stock (WSU 79-1146, ATCC^®^) was propagated in CRFK cells. Viral titers were determined on CRFK cells using Reed and Muench’s method [19] and reported as median 50 tissue culture infectious dose (TCID_50_)/mL. The virus titer was 10^7^ TCID_50_/mL, and the virus stock was aliquoted and stored at −80°C for further experiments as described previously [17, 18].

### Compound preparations

The antiviral activities of flavonoids derived from plant sources have been described for their effectiveness, which are included in this study and summarized in Table-1 [20–43]. All compounds (purity >98%–99%) were purchased from ChemFaces (Wuhan, China), and the compound powders were dissolved in 100% dimethyl sulfoxide (DMSO, Sigma-Aldrich, St. Louis, MO, USA) to a concentration of 10 mM stock solution. The dissolved compounds were kept at −25°C until further use. The compounds were prepared at the indicated concentrations in cell culture medium for use in all subsequent experiments.

**Table-1:** List of flavonoids against viral infection used in this study.

Bioactive flavonoid	Class	Plant source(s)	Antiviral activity	References
Apigenin (PubChem CID: 5336254)	Flavone	*Scutellaria indica L*	Influenza A (*in vitro*: EC_50_ = 5–20 µM) Dengue virus (*in vitro*: IC_50_ = 8.74 ± 0.08 µM)	[20, 21]
Baicalin (PubChem CID: 64982)	Flavone	*Scutellaria baicalensis* Georgi., *Thalictrum baicalense*	SARS-CoV-2 (*in vitro*: EC_50_ = 8.0–9.0 µM) Marek’s disease virus (Virucidal effect at 20 µg/mL)	[22, 23]
Diosmin (PubChem CID: 5281613)	Flavone	*Scrophularia nodosa*	African swine fever virus (*in vitro*: 40% inhibition at 10 µg/mL)	[24]
Linarin (PubChem CID: 5317025)	Flavone	*Silene firma, Scoparia dulcis, Uncaria sinensis* (Oliv.) Havil.	Duck hepatitis A virus (*in vitro*: 69.3% inhibition at 20 µg/mL)	[25]
Luteolin (PubChem CID: 5280445)	Flavone	*Dracocephalum ruyschiana L., Verbascum lychnitis, Carex fraseriana*	Influenza A virus (*in vitro*: EC_50_ = 6.89–7.15 µM) Respiratory syncytial virus (*in vitro*: 2.08–24.71 µM; *in vivo*: 50 mg/kg) SARS-CoV-2 RdRp (*in vitro*: IC_50_ = 4.6 ± 0.3 µM)	[26–28]
Hesperidin (PubChem CID: 10621)	Flavanone	*Citrus sinensis, Ficus erecta* var. *beecheyana, Myrtus communis*	Influenza A virus (*in vitro*: viral titer reduction at 3 log_10_ TCID_50_/0.1 mL)	[29]
Neohesperidin (PubChem CID: 442439)	Flavanone	*Citrus aurantium* L.	SARS-CoV-2 Spike protein (*in silico*: binding affinity at –60.95 kcal/mol)	[30]
Rutin (PubChem CID: 5280805)	Flavonol	*Styphnolobium japonicum* (L.) Schott.	SARS-CoV-2 3CL^pro^ (*in silico*: binding affinity at –9.2 kcal/mol)	[31]
Tiliroside (PubChem CID: 5320686)	Flavonol	*Agrimonia pilosa* Ledeb.	SARS-CoV 3a channel protein (*in vitro*: IC_50_ = 20 µM)	[32]
Quercetin (PubChem CID: 5280343)	Flavonol	*Sophora flavescens* Ait.	SARS-CoV-2 RdRp (*in vitro*: IC_50_ = 6.9 ± 1.0 µM)	[28]
Amentoflavone (PubChem CID: 5281600)	Biflavonoid	*Ginkgo biloba* L.	Coxsackievirus B3 (*in vitro*: EC_50_ = 25 ± 1.2 µM; virucidal: IC_50_ = 19–41 µg/mL) Herpes simplex virus type 1 (*in vitro*: EC_50_ = 11.11–28.22 µM)	[33, 34]
Isoginkgetin (PubChem CID: 5318569)	Biflavonoid	*Ginkgo biloba* L.	Foot-and-mouth disease virus (*in vitro*: EC_50_ =1.99 µM) SARS-CoV-2 (*in vitro*: EC_50_ = 22.80 µM)	[35, 36]
Puerarin (PubChem CID: 5281807)	Isoflavone	*Pueraria lobata*	Influenza A virus (*in vitro*: EC_50_ = 52.06 µM)	[37]
Epigallocatechin gallate (PubChem CID: 65064)	Flavanol-3-ols	*Acacia catechu* L.F*.*	Zika virus (*in vitro*: EC_50_ = 21.4 µM) Hepatitis B virus (*in vitro*: EC_50_ < 10µM) SARS-CoV-2 3CL^pro^ (*in vitro*: IC_50_ 7.58 µg/mL)	[38–40]
Epicatechin-3-O-gallate (PubChem CID: 65056)	Flavanol-3-ols	*Acacia catechu* L.F*.*	Herpes simplex virus type 1 (*in vitro*: EC_50_ = 4 µM)	[41]
Gallocatechin Gallate (PubChem CID: 5276890)	Flavanol-3-ols	*Corylus, Anacardium occidentale, Celastrus orbiculatus*	Enterovirus A71 (*in vitro*: 56% virus reduction at 10 µM Human immunodeficiency virus reverse transcriptase (*in vitro*: EC_50_ = 3.5 µM)	[42, 43]

EC_50_=50% maximal effective concentrations, RdRp=RNA-dependent RNA polymerase, SARS-CoV-2=Severe acute respiratory syndrome coronavirus, 3CL^pro^=3-Chymotrypsin-like cysteine protease, TCID_50_=50 tissue culture infectious dose, IC_50_=Half-maximal inhibitory concentration

### Cell cytotoxicity and cell viability

Crandell–Rees feline kidney cells were grown on 96-well plates at a concentration of 1.8 × 10^5^ cells/well and incubated at 37°C in a humidified incubator with 5% CO_2_ overnight. The compounds were diluted in MEM with 2% FBS 200, 100, 50, 25, 10, 1, and 0.1 μM and incubated with the cells at 37°C in a humidified incubator with 5% CO_2_ for 24 h. To determine cytotoxicity, cell viability was evaluated using the CellTiter 96 Aqueous non-radioactive cell proliferation assay (MTS assay, Promega, Madison, WI, USA), as described [17, 18]. Briefly, in the MTS assay, tetrazolium dye reduction, due to cellular metabolism, forms the formazan product, which changes the color of the media from yellow to brown. Color intensity is proportional to the number of living cells and is determined by measuring optical density at 490 nm on a multimode reader (Synergy H1 Hybrid Multi-Mode Reader, BioTek^®^, USA). Data were normalized with that of the untreated cell control, which was defined as 100%. Furthermore, 10% and 50% cytotoxicity concentrations (CC_10_ and CC_50_) were calculated as the concentration required to reduce cell viability by 10% and 50%, respectively.

### Viral kinetics of FIPV in CRFK cells

In a 24-well plate, 2 × 10^5^ CRFK cells/well were infected with FIPV at 50 TCID_50_ per well in a 37°C humidified incubator with 5% CO_2_ for 2 h for viral adsorption. The inoculum was removed, and the cells were maintained in MEM with 2% FBS. The infected cells were harvested at different time points: 0-, 2-, 4-, 6-, 8-, 10-, 12-, and 24-h post-infection (HPI). Feline infectious peritonitis virus replication kinetics at each time point was determined by measuring intracellular and extracellular viral RNAs with RT-qPCR.

### Cell-based antiviral activity assay

**Figure-1:**
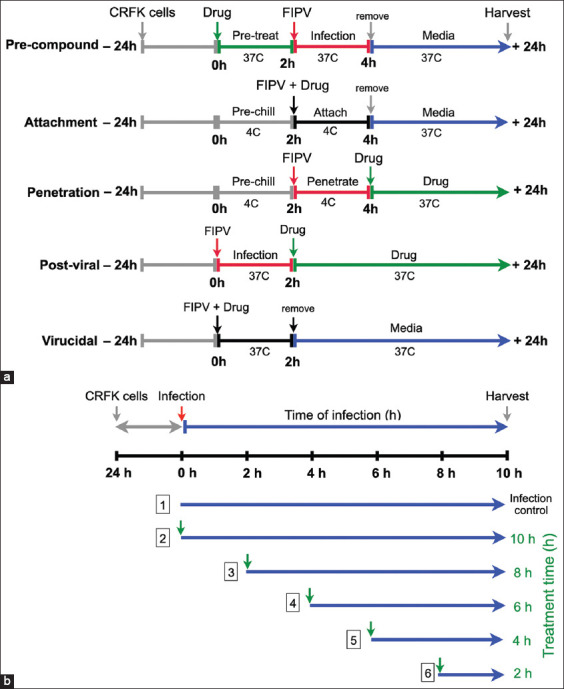
(a) Schematic representation of the different treatment approaches. Antiviral activity assays of flavonoids were performed according to the virus life cycle and steps of infection, including pre-compounds treatment, attachment, penetration, post-viral infection and virucidal assays. (b) Experimental timelines and antiviral effects in the time-of-addition assay. The feline infectious peritonitis virus-infected Crandell–Rees feline kidney cells were treated with the compounds at different time points after post-viral infection (0, 2, 4, 6 and 8 h post-infection; green arrows).

Crandell–Rees feline kidney cells were seeded into a 96-well plate at 1.8 × 10^4^ cells/well and cultured in MEM containing 7% FBS. Pre-compound, attachment, penetration, post-viral entry, and virucidal assays were performed based on each step of viral infection. All assays were performed using various concentrations of flavonoids: 100, 50, 25, 10, 1, and 0.1 μM/well with FIPV-infected CRFK cells. For the antiviral activity assay, FIPV was used at 50 TCID_50_/well in all experiments. Crandell–Rees feline kidney cells were inoculated with FIPV and treated with 0.1% DMSO as vehicle control. GC376 (TargetMol®, Wellesley Hills, MA, USA) was used as the FIPV-specific drug control [17, 18]. Three experiments were performed in duplicate wells for each compound. The conditions of the antiviral activity assay are shown in Figure-1a.

### Pre-compound assay

To determine the prophylactic effect of the flavonoids, CRFK cells were incubated with various concentrations of compounds at 37°C in a humidified incubator with 5% CO_2_ for 2 h. The cells were washed twice with phosphate-buffered saline (pH 7.4) (PBS, Invitrogen, Carlsbad, CA, USA) and inoculated with FIPV at 37°C for 2 h for viral adsorption. The cells were washed and maintained with MEM containing 2% FBS at 37°C in a humidified incubator with 5% CO_2_ for 24 h. The cytopathic effect (CPE) of the treated and infected cells was observed under an inverted microscope (Olympus CKX41, Tokyo, Japan).

### Attachment assay

To determine whether the compounds could interfere with virus binding to the host cells, CRFK cells in a 96-well plate were prechilled at 4°C for 1 h, and cotreated with the FIPV inoculum and the diluted compounds or controls at 4°C for 1 h to allow virus binding, but not entry to the host cells. The supernatants were subsequently removed, and the cells were gently washed with cold PBS and maintained in MEM with 2% at 37°C in a humidified incubator with 5% CO_2_ for 24 h.

### Penetration assay

To determine whether the compounds could interfere during virus penetration after binding to the host cells, CRFK cells in a 96-well plate were prechilled at 4°C for 1 h and inoculated with FIPV inoculum at 4°C for 1 h to allow virus binding to the cells. After 1 h for viral adsorption, the cells were shifted to 37°C for virus penetration into the cells, and then cotreated with the compounds in MEM with 2% at 37°C in a humidified incubator with 5% CO_2_ for 24 h.

### Post-viral entry assay

To determine whether the compounds could inhibit viral replication and translation in the host cells after infection, CRFK cells were inoculated with FIPV at 37°C for 2 h for viral adsorption. The infected cells were washed with PBS, and subsequently treated with various concentrations of compounds in MEM with 2% at 37°C in a humidified incubator with 5% CO_2_ for 24 h.

### Cotreatment assay

To determine whether the compounds could deactivate or destroy viruses before viral infection to the host cells, FIPV inoculum was exposed to various concentrations of the compounds. The mixture of FIPV and compounds was incubated with the cells at 37°C in a humidified incubator with 5% CO_2_ for 2 h. The cells were washed with PBS and maintained in MEM with 2% FBS at 37°C in a humidified incubator with 5% CO_2_ for 24 h.

### Time-of-addition (ToA) assay

This time-based approach of drug-addition determined how compounds inhibit the virus in the early or late steps of FIPV post-infection before losing their antiviral activities in cell culture. The compounds that inhibited viral replication in the post-viral entry assay were subjected to the ToA assay (Figure-1b). In a 24-well plate, 2 × 10^5^ CRFK cells/well were incubated with FIPV at 50 TCID_50_ at 37°C in a humidified incubator with 5% CO_2_ for 2 h for viral adsorption. The cells were washed with PBS to remove excess virus particles. The compounds at the maximum dose of non-cytotoxic concentrations were incubated with the infected cells for different time points (at 0-, 2-, 4-, 6-, and 8-HPI) after FIPV infection in separate duplicates, and then the cell culture plates were incubated at 37°C in a humidified incubator with 5% CO_2_. At 10-HPI, the cells were harvested for a viral production study by RT-qPCR.

### Virucidal effect of flavonoids at different time points

The compounds that showed antiviral activity in the cotreatment assay were evaluated. Feline infectious peritonitis virus inoculum at 50 TCID_50_/mL was directly incubated at the maximum concentrations of the compounds that were non-cytotoxic at 37°C in a 1.5 mL microcentrifuge tube for 0, 15, 30, 60, and 120 min. After the indicated incubation, the mixtures of virus and compounds were immediately subjected to viral titration according to Reed and Muench’s method [19]. Viral titers in compound-treated wells were compared with the untreated control.

### Cytopathic effect and detection of the positive FIPV-infected cells using the immunoperoxidase monolayer assay (IPMA)

For initial screening, FIPV infection was determined using crystal violet staining for all antiviral assays. Briefly, the supernatants were removed from the compound-treated and FIPV-infected CRFK and control cells, and the cells were fixed with cold methanol at room temperature (25°C) for 30 min. Fifty microliters of crystal violet solution (0.5% crystal violet in 80% water and 20% methanol) were added into each well, and the culture plate was rocked gently at 25°C for 30 min. The staining solution was removed and washed with plenty of water. Cytopathic effect was observed, characterized by multinucleated giant and rounded cells, under an inverted microscope (Olympus CKX41). To demonstrate FIPV antigens, compound-treated and FIPV-infected CRFK cells and control cells were subjected to IPMA, as described by previous studies [17, 18]. Briefly, the cells were fixed with cold methanol at 25°C for 30 min and washed with PBS with 0.1% Tween (PBST). The fixed cells were incubated with primary antibody (mouse anti-FIPV3-70, dilution 1:500, ThermoFisher, Carlsbad, USA) at 37°C in a moist chamber for 1 h and washed with PBST before incubation with goat anti-mouse IgG-HRP antibody (dilution 1:800, ThermoFisher), at 37°C in a moist chamber for 1 h. The FIPV antigens were visualized by adding the DAB substrate (DAKO, Santa Clara, USA), the brown color of which was developed in the cytoplasm of the infected cells. Five to ten images of positive FIPV-infected cells were recorded at a magnification of 100× using a phase-contrast inverted microscope (Olympus IX73). Image analysis was used to calculate the positive signals of the infected cells using CellProfiler version 4.1.3 (Board Institute, Cambridge, MA, USA), as described [17, 18]. The data were normalized as percentages of positive infection and normalized to those of cell controls; the non-infected cells had 0% infection, and the untreated, infected cells had 100% infection. Finally, 50% maximal effective concentrations (EC_50_) and 90% maximal effective concentrations (EC_90_) of the compounds were calculated as the concentrations required to reduce viral infection by 50% and 90%, respectively.

### Absolute quantification of FIPV RNA using RT-qPCR

Intracellular and extracellular FIPV from infected CRFK cells was determined using RT-qPCR, as described [17, 18]. The viral RNAs were isolated from the infected cells using Trizol reagent (Invitrogen), and then clarified using Direct-zol RNA MiniPrep (Zymo Research Corporation, Tustin, CA, USA). One microgram of total RNA was reverse transcribed into cDNA using RevertAid reverse transcriptase enzyme and random hexamers (Thermo Fisher Scientific Inc.). The primers specific to FCoV 3′UTR were 5’–GGC AAC CCG ATG TTT AAA ACT GG–3’ (forward) and 5’–CAC TAG ATC CAG ACG TTA GCT C–3’ (reverse) [17, 18]. For qPCR amplification, the qPCR reaction contained 5 μL iTaq Universal SYBR Green Supermix (2×) (Bio-Rad Laboratories, Hercule, CA, USA), 2 μL cDNA template, and 0.5 μL of forward and reverse primer each. The qPCR condition included an initial denaturation of DNA at 95°C for 30 s; 30 cycles of 5 s at 95°C of denaturation and 5 s at 60°C of annealing and extension, followed by a melting curve analysis from 65°C to 95°C, with 0.5°C increment, according to the C1000 Touch thermal cycler (Bio-Rad Laboratories). A standard curve of viral copy numbers was generated from the plasmid containing the FIPV 3′UTR sequence [17, 18] by 10-fold serially diluting the plasmid from 10^−1^ to 10^−7^ plasmid molecules/μL and using it for viral load quantification. Quantitative polymerase chain reaction was performed from two biological replicates and three independent experiments were performed.

### Statistical analysis

After data normalization, the dose-response curves were generated by fitting the non-linear regression equations derived from the normalized data of the cell-based antiviral activity assay, including pre-compound, penetration, post-viral entry, and cotreatment assays. The CC_50_ and EC_50_ values were calculated based on the dose-response curve using GraphPad version 9.4.1 (Prism, San Diego, CA, USA). For the ToA and virucidal assays, data were analyzed using a one-way analysis of variance when they were significantly different, *post hoc* comparison with the Tukey test method (GraphPad version 9.4.1, Prism) was conducted. The selective index (SI) was calculated by dividing CC_50_ by EC_50_ to demonstrate compound efficacy. All results are presented as mean and SD. The significant level for analysis was at p < 0.05.

## Results

### Cell viability assay analysis

**Figure-2:**
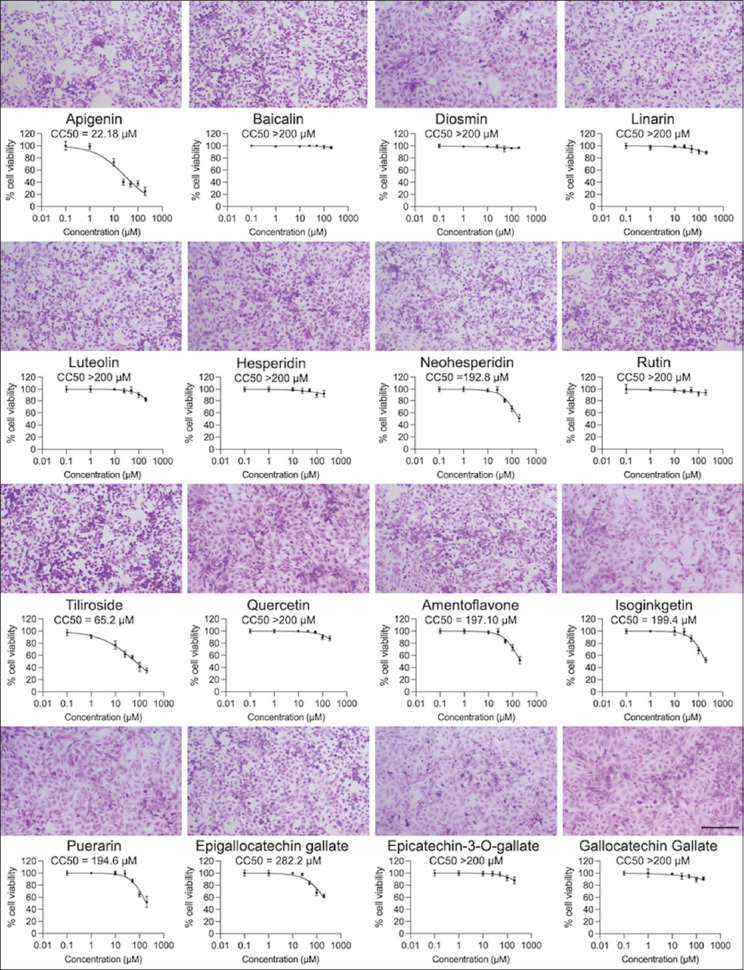
Cytotoxicity of sixteen flavonoids on Crandell–Rees feline kidney cells at 24 h post-infection. Crandell–Rees feline kidney cells treated with each flavonoid at 100 μM were stained with 0.5% crystal violet to reveal viable cells (upper panels). The scale bar is 100 μm. The cytotoxicity was measured using MTS assay (lower panels). The 50% cytotoxicity concentrations values were calculated using non-linear regression analysis. Bars present mean and standard deviation values.

The cytotoxicity assay confirmed that the concentration of the flavonoid compounds up to 100 μM was non-toxic to CRFK cells. The analysis of cell viability demonstrated a gradually decreasing trend after the treatment with increasing compound concentrations. Apigenin and tiliroside had mild cytotoxicity, with CC_50_ value of 22.18 μM and 65.20 μM using the MTS assay, respectively. We quantified cell abundance based on the observation of crystal violet staining. The results from the MTS assay and crystal violet staining were consistent (Figure-2). Therefore, the non-cytotoxic compounds were subjected to antiviral activity assays.

### Viral kinetics of FIPV

**Figure-3:**
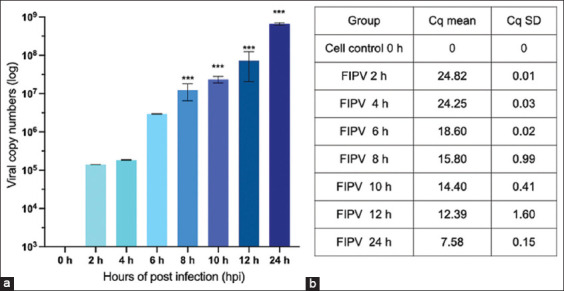
(a) Feline infectious peritonitis virus (FIPV) replication kinetics in Crandell–Rees feline kidney cells. The cells were infected with FIPV at 50 tissue culture infectious dose/well. The intracellular and extracellular FIPVs in the culture wells were measured by quantitative reverse transcription-polymerase chain reaction at different time points after post-infection. (b) The viral copy number of the replicated viruses was determined from the average of three independent experiments. Quantitative cycles of FIPV replication kinetics were presented as means and standard deviations. Differences of viral kinetics by time point were analyzed using analysis of variance (***p < 0.001).

Feline infectious peritonitis virus at 50 TCID_50_ that infected CRFK cells in each well induced a significant degree of CPE, including multinucleated giant cells and rounded cells at 24-HPI. In FIPV-infected cells, the viral RNA slightly increased at the early stage of infection between 2- and 4-HPI, and an exponential increase was found between 8- and 12-HPI. The total amount of viral RNAs peaked at 24-HPI. Therefore, the antiviral efficacy of flavonoids against FIPV replication was evaluated based on viral titers at 24-HPI, whereas the inhibitory effect during the early stage of viral infection was evaluated at 10-HPI. The replication kinetics of FIPV is shown in Figure-3.

### Flavonoids neither possess the cell protection effects nor interfere with FIPV binding and entry in CRFK cells

Flavonoids can inhibit the replication of many viruses by targeting various viral proteins with different efficacies. Hence, we sought to clarify the effects of flavonoids on different steps of virus infection. To achieve this, we performed experiments using different approaches in a broad dilution range of compounds. For the cell protection effect, we pretreated cells with flavonoids for 2 h, and then FIPV was absorbed for 2 h. After 24-HPI, the viral titers were determined by using crystal violet staining and IPMA. The results showed that all flavonoids were not sufficient to protect the cells from viral infection. To study whether flavonoids affected FIPV binding to the viral receptor on CRFK cell surface, flavonoids and FIPV were co-adsorbed on CRFK cells at 4°C for 1 h for viral attachment, which allowed the virus to bind the cell receptor, but not enter the cells. Next, unabsorbed FIPV was removed, and the cells were transferred to 37°C to permit FIPV internalization. After 24-HPI, all flavonoids did not interfere with FIPV binding to the cells. Moreover, we investigated the effects of flavonoids during FIPV penetration into the cells by incubating FIPV with CRFK cells at 4°C for 1 h. The unbound viral particles were subsequently removed, and the cells were washed with PBS. Then, the flavonoids were added to the cells before they were transferred to the 37°C incubator for virus penetration and entry for 24 h. The penetration assay data showed that the flavonoids did not affect FIPV penetration into the cells. As expected, the FIPV-infected CRFK cells treated with GC376, a 3CL protease inhibitor, did not possess the prophylactic and virus attachment effects at a high concentration, suggesting the sufficient removal of the compounds. In the penetration assay (37°C), GC376 inhibited FIPV replication at high concentrations of up to 50 μM.

### Isoginkgetin and luteolin potently inhibit FIPV replication post-viral entry

**Figure-4:**
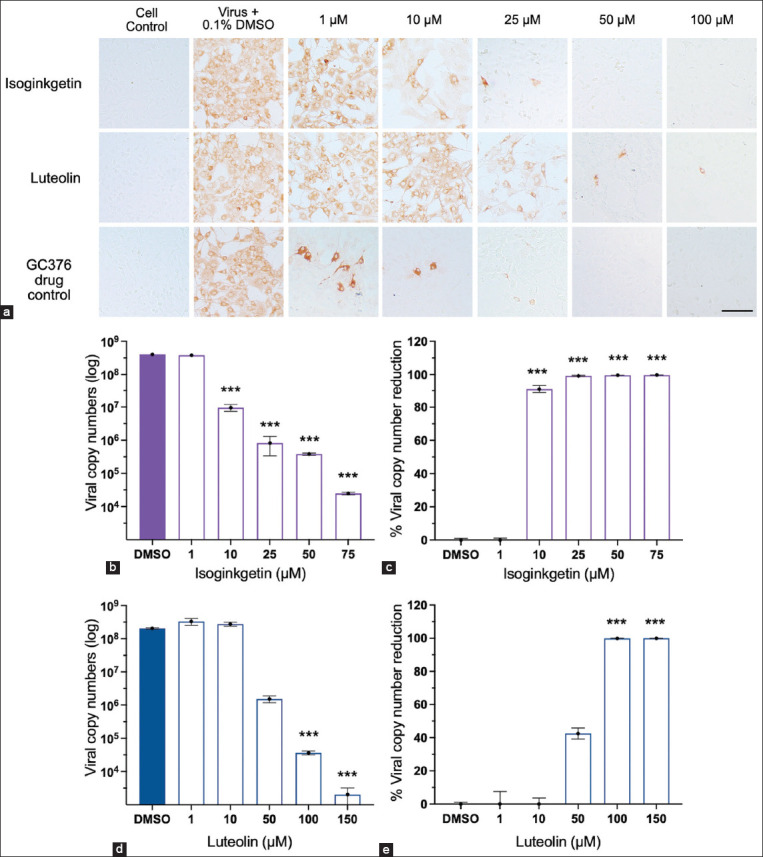
(a) Antiviral activity assay of isoginkgetin and luteolin. Both compounds could exert their inhibitory effects after feline infectious peritonitis virus (FIPV) infection for 2 h before the treatment, when compared to the positive drug control (GC376). 0.1% dimethyl sulfoxide (DMSO) in Crandell–Rees feline kidney (CRFK) cells was included as a negative control. The scale bar is 100 μm. (b and c) Viral loads of infected CRFK cells treated with isoginkgetin and (d and e) luteolin with a dose-dependent manner were quantified by reverse transcription-quantitative polymerase chain reaction. (b and d) The data were presented as quantification of viral copy numbers and (c and e) percentage of viral reduction, when compared to those of virus treated with 0.1% DMSO group. Differences between the flavonoid-treated and the FIPV-infected CRFK cells in a dose-dependent manner compared to virus control were assessed using analysis of variance (***p < 0.001).

To determine whether flavonoids could inhibit FIPV replication after infection, we investigated the antiviral activity of flavonoids at various compound dilutions. Crandell–Rees feline kidney cells were infected FIPV at 37°C for 2 h. Then, the unabsorbed inoculum was removed and replaced with the flavonoids in fresh media. After 24-HPI of treatment, compound-treated and FIPV-infected cells were observed for CPE and FIPV antigens using IPMA. Compared to the control, two (isoginkgetin and luteolin) out of 16 flavonoids showed a dose-dependent inhibition on FIPV replication (Figure-4a). The EC_50_ values evaluated using IPMA for isoginkgetin and luteolin were 4.77 ± 0.09 μM (SI = 41.80) and 36.28 ± 0.03 μM (SI = 6.88), respectively (Table-2). In addition, epigallocatechin gallate and amentoflavone could inhibit 50% of FIPV replication at high concentrations (Table-2). Hence, we conducted dose-response profiling for isoginkgetin and luteolin based on RT-qPCR analysis. A noticeable reduction in intracellular and extracellular viral RNAs was demonstrated in isoginkgetin and luteolin treatments. A significant reduction in FIPV RNAs was observed after isoginkgetin treatment at 10 μM, at which the inhibition of viral copy numbers was >90%, whereas luteolin required a high concentration for reducing FIPV replication. The RT-qPCR data corresponded to the IPMA findings in a dose-dependent manner (Figures-4b–e). These results indicate that isoginkgetin and luteolin were more likely to exert their antiviral activity at a post-viral entry step.

**Table-2:** The cytotoxicity (CC_50_ and CC_10_) in CRFK cells, the effective concentration, (EC_50_ and EC_90_), and the selective indices of flavonoids against FIPV-infected CRFK cells during post-viral entry.

Compound	Cytotoxicity		Antiviral activity		Selective index
		
	CC_50_^1^; µM	CC_10_^2^; µM	EC_50_^3^; µM	EC_90_^4^; µM	CC_50_/EC_50_^5^
Luteolin	248.0 ± 3.40	136.80 ± 2.37	36.28 ± 0.03	65.98 ± 1.82	6.83
Isoginkgetin	199.40 ± 0.33	77.43 ± 2.52	4.77 ± 0.09	28.82 ± 1.46	41.80
Epigallocatechin gallate	282.20 ± 0.12	84.98 ± 1.93	79.07 ± 0.01	196.50 ± 1.96	3.57
Amentoflavone	197.10 ± 2.29	155.50 ± 2.19	144.10 ± 0.01	242.90 ± 2.40	1.37

1 CC_50_: Compound concentration required to reduce cell viability by 50% as determined by MTS assay,

2 CC_10_: Compound concentration required to reduce cell viability by 10% as determined by MTS assay,

3 EC_50_: Compound concentration required to effectively protect 50% of cells from virus infection as determined by IPMA,

4 EC_90_: Compound concentration required to effectively protect 90% of cells from virus infection as determined by IPMA,

5 Selective index: A ratio by CC_50_/EC_50_, Data values represent as mean ± SD of three independent experiments, EC_50_=50% maximal effective concentrations, EC_90_=90% maximal effective concentrations, CC_50_=50% cytotoxicity concentrations, CC_10_=10% cytotoxicity concentrations, FIPV=Feline infectious peritonitis virus, CRFK=Crandell–Rees feline kidney

**Figure-5:**
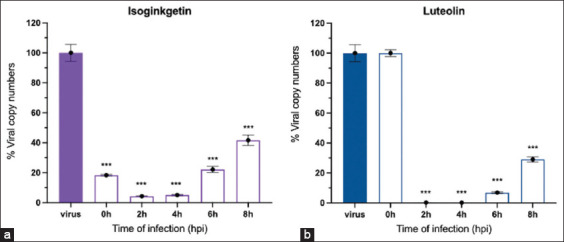
(a and b) Antiviral effects were determined according to viral load reduction of time-of-addition treatment compared to that of virus control. Percentage of viral copy numbers in the isoginkgetin and luteolin treatment groups added at different time points are presented with bar graphs. Bars represent mean and standard deviation values from three independent experiments using analysis of variance (***p < 0.001).

Because of post-viral entry inhibition of isoginkgetin and luteolin, we examined their antiviral mechanisms in a time-course assay. To identify the key step of viral infection affected by isoginkgetin and luteolin, ToA was performed to identify the most drug-sensitive phase of FIPV replication at 0-, 2-, 4-, 6-, 8-, and 10-HPI (Figure-1b). Fixed concentrations of isoginkgetin (28.82 μM) and luteolin (65.98 μM), based on the EC90 values, at which cellular toxicity was not observed, were used. The treatment was given to the infected cells at the indicated times, and then both flavonoids remained in the wells until 10-HPI. The viral RNAs were quantified by RT-qPCR to determine the viral load. The ToA findings were performed to confirm that isoginkgetin had a major inhibitory effect immediately after FIPV internalization (at 0-HPI, Figure-5a). Isoginkgetin showed the maximal efficiency at 2–4-HPI; its inhibitory efficiency decreased after 6-HPI, which was suggested by the increased progeny virus yield. Therefore, the ToA result revealed that isoginkgetin was rapidly absorbed and immediately reached effective potency inside the cells. At 2–4-HPI, luteolin demonstrated a significant inhibition of FIPV replication, whereas at 8-HPI, luteolin moderately reduced FIPV RNA in infected cells (Figure-5b). Thus, these data show that isoginkgetin and luteolin effectively inhibited the proliferation of FIPV at the early step of the replication cycle.

### Isoginkgetin exhibited an extracellular virucidal effect against FIPV

To determine the effective time needed for flavonoids to exert their virucidal effects on extracellular FIPV particles, we coincubated flavonoids and FIPV inoculum at 37°C for 2 h, removed the supernatant, added fresh media, and incubated at 37°C for 24 h. Isoginkgetin could exert a direct extracellular virucidal effect on FIPV replication in a dose-dependent manner. The EC_50_ value determined by IPMA was 40.62 ± 1.50 μM (SI = 4.90), which is greater than the EC_50_ of the post-viral entry experiment by approximately 10-fold.

**Figure-6:**
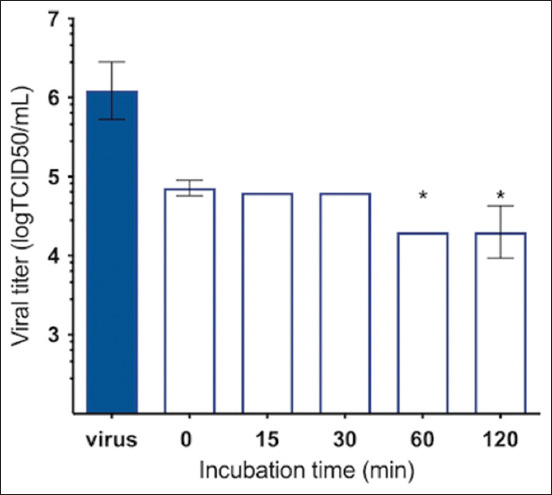
Virucidal effect of isoginkgetin incubated with feline infectious peritonitis virus at 50 tissue culture infectious dose (TCID_50_)/well at different time points: 0, 15, 30, 60, and 120 min, respectively, compared to the untreated control. Viral titers were subsequently tested by a TCID_50_ assay. Bars represent mean and standard deviation values. The virucidal effects of isoginkgetin at different time points were evaluated using analysis of variance (*p < 0.05).

To investigate the shortest time required by isoginkgetin to exert its virucidal effects, the experiment was performed at different coincubation times (0, 15, 30, 60, and 120 min) with isoginkgetin at the maximum concentration of 75 μM. At this concentration, cell viability was at least 90%. Feline infectious peritonitis virus was directly incubated with isoginkgetin at the maximum concentration. After the indicated times of incubation, the mixtures were 10-fold serially diluted, and viral titers were evaluated and reported as TCID_50_/mL. Isoginkgetin significantly inactivated FIPV by decreasing 2 and 1.89 log_10_TCID_50_/mL at 60 and 120 min (p < 0.05), respectively, compared with the untreated control (Figure-6). These data suggest that isoginkgetin is virucidal and reduces viral infection.

## Discussion

Feline infectious peritonitis is a fatal infectious disease in young cats, with high mortality, and is caused by FIPV. Several vaccines have been developed; however, they do not provide adequate immune protection. An effective drug that can inhibit FIPV and reduce disease severity is required [1–3, 9]. Flavonoids are bioactive compounds found in natural products, particularly in plants, and possess less toxicity at high doses. Therefore, flavonoids can be an alternative or supportive treatment for FIP in cats. We examined the antiviral effects of flavonoids on different stages of viral replication. Collectively, this study has shown that flavonoids, such as isoginkgetin and luteolin, exhibit robust antiviral abilities by interfering with the early stage of FIPV infection.

Isoginkgetin is a member of the natural bioflavonoid constituent in *Ginkgo biloba*, which is a derivative of amentoflavone. Studies have reported a broad range of biological properties, including pre-mRNA splicing inhibition, inhibition of matrix metalloproteinase 9 production, and inhibition of 20S proteasome leading to activated lysosome regulator to autophagic machinery [44–46]. Isoginkgetin contains the 4H-chromen-4-one scaffold, which possesses pharmacological properties [36]. Recently, the antiviral activity of isoginkgetin on severe acute respiratory syndrome CoV-2 (SARS-CoV-2) was demonstrated in infected Vero cells [36]. The EC_50_ value against SARS-CoV-2 was 22.81 μM, which was higher than that in our study (EC_50_ = 4.77 ± 0.09 μM). This suggests that isoginkgetin exhibited better inhibition of FIPV replication. Moreover, the predicted molecular binding of isoginkgetin to a key enzyme of CoVs, including a 3-chymotrypsin-like cysteine protease (3CL^pro^), was performed. Isoginkgetin revealed a good binding affinity to the active site of 3CL^pro^, with a binding affinity of −9.38 and −8.9 kcal/mol [36, 47]. Isoginkgetin could inhibit foot-and-mouth disease virus (FMDV) during post-viral entry, with an EC_50_ value of 1.93 ± 0.21 μM. In addition, isoginkgetin could inhibit FMDV 3C^pro^ activity with a half-maximal inhibitory concentration (IC_50_) value of 39.03 μM [35]. Foot-and-mouth disease virus 3C^pro^ is a chymotrypsin-like cysteine protease, which processes viral polyproteins for the virus life cycle as well as interact with 3CL^pro^ of CoVs [35]. These studies confirm that isoginkgetin inhibits viral proteases of CoVs and picornaviruses. Thus, the inhibition mechanisms of this compound on FIPV 3CL^pro^ activity must be evaluated. In addition to its antiprotease effects, isoginkgetin acts as a virucidal when the virus and compounds are cotreated before incubating with cells (EC_50_ = 2.01 ± 0.07 μM) [35], which corresponds to the virucidal effect on FIPV in our study. Isoginkgetin exerts antiviral activity by inactivating the virus at a high concentration, possibly by interfering with lipid envelope stability [35, 38]. In the virucidal assay, isoginkgetin was stable and effective on FIPV upon inactivation for 120 min; therefore, it could reduce secreted viral particles on the material surface. However, the precise mechanism of isoginkgetin on viral inactivation remains to be elucidated.

In this study, three flavonoids demonstrated antiviral activity during post-viral entry. Luteolin is a flavone found in a variety of fruits and vegetables and has been reported to contain potential pharmacological effects, such as anti-inflammatory, antioxidant, and antitumor properties [15, 16]. Our results revealed its antiviral effect against FIPV replication. Similar results have been shown for influenza A virus [26], respiratory syncytial virus [27], and FMDV [35]. Luteolin had an inhibitory activity against SARS-CoV-2 RdRp with an IC_50_ value of 4.6 μM [28]. In addition, a luteolin derivative, 7-methylluteolin, was found to be effective against FIPV 3CL^pro^ with an IC_50_ value of 28.5 ± 4.2 μM [17]. Luteolin could exert its antiviral activity through CoV proteins during viral infection. Amentoflavone, a prototype of isoginkgetin, possesses a broad-spectrum antiviral activity against various viruses [24, 47, 48]. However, our results showed that a high concentration of amentoflavone was required to inhibit FIPV replication. Amentoflavone derivatives (e.g., isoginkgetin) showed better interaction with SARS-CoV-2 3CL^pro^ [48]. In our study, a high concentration of epigallocatechin gallate was required for post-viral inhibition, which is greater than that for HIV-1 and hepatitis B virus [39, 43]. To the best of our knowledge, this is the first report on the antiviral effects of isoginkgetin and luteolin against FIPV replication during post-viral entry. Moreover, isoginkgetin exerts virucidal activity against extracellular FIPV particles. Recently, molnupiravir, a drug under emergency use authorization by the FDA to treat COVID-19, has been used in unresponsive or recurrent FIP following GS- and GC-based therapies in both injectable and oral forms. However, adverse effects were found in some cats with skin conditions, seizures, or severe leukopenia [49]. Thus, insufficient purity and concentration as well as drug-induced viral resistance of antiviral agents remain a concern. Therefore, this finding for new antiviral agents from other sources, such as nature-derived products, maybe another attractive therapeutic option for FIP treatment in cats.

## Conclusion

We demonstrated the *in vitro* antiviral effects of flavonoids against FIPV in CRFK cells. Isoginkgetin interfered with intracellular virus replication at the early stage besides an inactivation of free FIPV particles. In addition, luteolin showed antiviral activity on post-viral entry. Studies on the mode of action of the flavonoids would help improve their efficacies and rationally design effective inhibitors for FIPV. The findings on the antiviral activity of isoginkgetin and luteolin could provide valuable information for developing effective antiviral agents with therapeutic safety for FIP treatment.

## Authors’ Contributions

ST and PL: Conceptualization. CT, VL, and NP: Methodology. CT, ST, VL, and AP: Data analysis. CT and ST: Visualization. ST and PL: Supervision and project administration. CT, ST, and PL: Writing, reviewing and editing. All authors have read, reviewed, and approved the final manuscript.
